# Supplementary material for: Mapping the evolution of fertility support policies in China: A content and instrumental analysis
Source: PLoS One. 2025 Oct 9;20(10):e0332137. doi: 10.1371/journal.pone.0332137 (PMC12510515; doi:10.1371/journal.pone.0332137)
Supplement: S1 Appendix — (ZIP) [file pone.0332137.s001.zip › S1 Appendix. 226 original policy documents/17-国家卫生和计划生育委员会办公厅关于印发流动人口卫生和计划生育基本公共服务均等化试点工作方案的通知(FBM-CLI-4-214788).doc]

国家卫生和计划生育委员会办公厅关于印发流动人口卫生和计划生育基本公共服务均等化试点工作方案的通知

**发布部门：** [国家卫生和计划生育委员会(已撤销)](https://www.pkulaw.com/law/chl?Aggs.IssueDepartment=60319) [机构沿革](javascript:void(0))

**发文字号：**国卫办流管发〔2013〕35号

**发布日期：**2013.11.28

**实施日期：**2013.11.28

**时效性：** [失效](https://www.pkulaw.com/law/chl?Aggs.TimelinessDic=02)

**效力级别：** [部门工作文件](https://www.pkulaw.com/law/chl?Aggs.EffectivenessDic=XE0303)

**法规类别：** [计划生育管理](https://www.pkulaw.com/law/chl?Aggs.Category=09705) [人才流动](https://www.pkulaw.com/law/chl?Aggs.Category=08806)

**失效依据：**[国家卫生计生委关于宣布失效第一批委文件的决定](javascript:TMP(288762,0,'chl'))

国家卫生和计划生育委员会办公厅关于印发流动人口卫生和计划生育基本公共服务均等化试点工作方案的通知
（国卫办流管发﹝2013﹞35号）

北京市、天津市、河北省、山西省、辽宁省、吉林省、黑龙江省、上海市、江苏省、浙江省、安徽省、福建省、山东省、河南省、湖北省、湖南省、广东省、广西壮族自治区、重庆市、四川省、贵州省、云南省、西藏自治区、陕西省、宁夏回族自治区、青海省、新疆维吾尔自治区卫生厅局、人口计生委（卫生计生委）：
　　《流动人口卫生和计划生育基本公共服务均等化试点工作方案》已经国家卫生计生委第12次主任会议讨论通过，现印发给你们，请认真组织实施。

国家卫生计生委办公厅
2013年11月28日

　　流动人口卫生和计划生育基本公共服务均等化试点工作方案

　　根据关于加强流动人口卫生计生工作的总体部署，为进一步落实流动人口卫生和计划生育基本公共服务，我委拟在原国家人口计生委等部门开展流动人口计划生育基本公共服务试点工作的基础上，在全国40个城市启动流动人口卫生和计划生育基本公共服务均等化试点工作。具体方案如下：
　　**一、**指导思想
　　以卫生计生机构改革为契机，按照国家基本公共卫生服务规范（2011版）和计划生育基本公共服务均等化的要求，坚持以人为本、保障基本、逐步均等、有序推进原则，完善和创新流动人口卫生和计划生育基本公共服务，大力推进流动人口卫生和计划生育基本公共服务均等化。

　　**二、**工作目标
　　在“十二五”期间，探索流动人口卫生和计划生育基本公共服务的工作模式和有效措施，促进流动人口卫生和计划生育信息共享与应用，提高流动人口卫生和计划生育基本公共服务可及性和水平，为建立流动人口卫生和计划生育基本公共服务制度积累经验。

　　**三、**试点范围
　　在全国27个省（区、市）40个流动人口较集中的城市开展试点工作（名单见附件）。

　　**四、**重点工作
　　（一）建立健全流动人口健康档案。为在辖区居住6个月以上的流动人口建立统一、规范的健康档案，及时掌握流动人口的健康状况。健康档案主要信息包括流动人口基本信息、主要健康问题及卫生服务记录等内容。流动人口健康档案应当及时更新。
　　（二）开展流动人口健康教育工作。在流动人口数量较多的社区、企业、厂矿、单位和学校等主要场所设置健康教育宣传栏和资料发放点，每年定期开展卫生和计划生育基本公共服务政策宣传活动，举办传染病防治等健康知识讲座，组织关爱流动人口健康义诊活动，提高流动人口健康素养，引导流动人口更好地接受服务。
　　（三）加强流动儿童预防接种工作。为辖区内居住满3个月的0-6岁流动儿童建立预防接种档案，采取预约、通知单、电话、手机短信、设立临时接种点等适宜方式，为流动适龄儿童及时建卡、接种。每年集中开展“查漏补种”活动，对漏种儿童及时补种。根据传染病防控需要，开展乙肝、麻疹、脊灰等疫苗补充免疫、群体性接种和应急接种工作。对入托入学流动儿童严格执行查验预防接种证等管理措施，不断提高流动适龄儿童疫苗接种率。
　　（四）落实流动人口传染病防控措施。对建筑工地、商贸市场、生产加工企业等流动人口密集地区，加强传染病监测工作，及时处置传染病疫情，切实落实流动人口艾滋病、结核病等传染病的免费救治等政策。推动城乡结合部环境卫生综合整治，改善流动人口居住环境。
　　（五）加强流动孕产妇和儿童保健管理。为流动孕产妇、儿童建立统一的保健管理档案。加强妇幼保健知识宣传。强化育龄妇女孕情监测、叶酸补服、流动孕产妇早孕建卡、孕期保健、高危筛查、住院分娩和产后访视等关键环节控制工作，保障母婴安全。完善0-6岁流动儿童家庭访视、定期健康检查、生长发育监测、喂养与营养指导等儿童保健服务。加强流动孕产妇及新生儿预防艾滋病、梅毒、乙肝母婴传播工作。
　　（六）落实流动人口计划生育基本公共服务。继续落实流动人口计划生育基本公共服务均等化试点工作的各项要求，全面开展计划生育法规政策宣传倡导、计划生育技术服务、优生优育、生殖健康、奖励优待等服务项目，重点落实国家规定的计划生育免费技术服务，为流动育龄人口提供避孕节育、优生优育科普宣传、免费发放避孕药具及健康指导服务。
　　（七）探索流动人口服务管理新机制。依托覆盖城乡的基层卫生服务机构和计划生育基层服务管理网络，创新工作模式和运行机制，提升服务能力和水平。探索卫生和计划生育基本公共服务覆盖流动人口的措施和路径，努力实现流动人口卫生和计划生育基本公共服务均等化工作有新突破。

　　**五、**工作要求
　　（一）加强组织领导，落实职责分工。试点省份要高度重视试点工作，特别是在机构改革过程中，要注重卫生计生部门协作配合，确保工作任务落实到位。试点城市要根据试点任务相应调整或成立工作协调小组，明确相关部门职责，建立联席会议制度，并抓紧组织编制试点实施方案，明确工作目标、主要任务、保障措施及进度安排，务求试点工作取得实效。
　　（二）增加经费投入，提供财政保障。中央财政继续安排资金支持试点工作。试点省份强化省级财政支出责任，参照当地户籍人口卫生和计划生育基本公共服务经费投入标准，将流动人口卫生和计划生育基本公共服务经费纳入当地公共财政支出预算范围予以保障。试点城市逐步加大经费投入，按照常住人口规模编制年度预算，使流动人口卫生和计划生育基本公共服务经费与需求相适应。
　　（三）开展调查研究，准确掌握情况。我委将于2014年第一季度组织开展试点城市流动人口卫生和计划生育基本公共服务现状调查工作，试点城市要按照统一要求，协助做好本地流动人口数据，特别是流动育龄妇女、孕产妇、0-6岁儿童等重点人群数据的清理核查工作，掌握本地区流动人口卫生和计划生育基本公共服务现状，为做好试点工作提供基础数据支持。
　　（四）整合信息资源，推动信息共享。试点城市要依托区域人口健康信息平台，完善居民电子健康档案，整合流动人口卫生和计划生育公共服务信息资源，普及应用居民健康卡，建立健全相关信息共享机制。完善现有的公共卫生服务相关信息系统和流动人口计划生育信息系统，逐步实现流动人口卫生和计划生育公共服务信息跨地区、跨部门的互联互通、共享应用。
　　（五）强化督导检查，完善考评机制。试点城市要将试点工作纳入基本公共服务绩效考核体系，定期开展监督检查。绩效考评结果要与财政补助挂钩。我委将建立试点工作通报制度，每年对试点工作组织开展督导评估，并根据检查评估结果动态调整试点地区和中央转移支付经费。各试点省份要在2013年底前完成对试点工作的具体部署和安排，并将相关情况于2014年1月底前报我委流动人口司。
　　附件
　　试点城市名单

| 序号 | 省（区、市） | 城市 | 序号 | 省（区、市） | 城市 |
| --- | --- | --- | --- | --- | --- |
| 1 | 北京 | 朝阳区 | 21 | 安徽 | 合肥 |
| 2 |  | 丰台区 | 22 | 福建 | 厦门 |
| 3 | 天津 | 滨海新区 | 23 |  | 泉州 |
| 4 |  | 津南区 | 24 | 山东 | 青岛 |
| 5 | 河北 | 石家庄 | 25 | 河南 | 郑州 |
| 6 | 山西 | 太原 | 26 | 湖北 | 武汉 |
| 7 | 辽宁 | 大连 | 27 | 湖南 | 长沙 |
| 8 | 吉林 | 长春 | 28 | 广东 | 深圳 |
| 9 | 黑龙江 | 哈尔滨 | 29 |  | 中山 |
| 10 | 上海 | 闵行区 | 30 | 广西 | 桂林 |
| 11 |  | 杨浦区 | 31 | 重庆 | 渝北区 |
| 12 |  | 松江区 | 32 | 四川 | 成都 |
| 13 |  | 宝山区 | 33 | 贵州 | 贵阳 |
| 14 | 江苏 | 南京 | 34 | 云南 | 玉溪 |
| 15 |  | 苏州 | 35 | 西藏 | 拉萨 |
| 16 |  | 无锡 | 36 | 陕西 | 西安 |
| 17 | 浙江 | 杭州 | 37 |  | 咸阳 |
| 18 |  | 宁波 | 38 | 宁夏 | 银川 |
| 19 |  | 嘉兴 | 39 | 青海 | 西宁 |
| 20 |  | 绍兴 | 40 | 新疆 | 克拉玛依 |

© 北大法宝：（[www.pkulaw.com](https://www.pkulaw.com/)) 专业提供法律信息、法学知识和法律软件领域各类解决方案。北大法宝为您提供丰富的参考资料，正式引用法规条文时请与标准文本核对。
欢迎查看所有[产品和服务](https://www.pkulaw.com/helps/2.html)。法宝快讯：[如何快速找到您需要的检索结果？](https://www.pkulaw.com/helps/70.html)[法宝](https://www.pkulaw.com/helps/69.html)V6有何新特色？

扫描二维码阅读原文

**原文链接：**<https://www.pkulaw.com/chl/1639eb2ba695acb1bdfb.html>
